# Lysophosphatidic Acid Signalling Regulates Human Sperm Viability via the Phosphoinositide 3-Kinase/AKT Pathway

**DOI:** 10.3390/cells12172196

**Published:** 2023-09-02

**Authors:** Hao-Yu Liao, Cristian O’Flaherty

**Affiliations:** 1 Department of Medicine, Experimental Medicine Division, Faculty of Medicine and Health Sciences, McGill University, Montreal, QC H3A 0G4, Canada; 2 Department of Surgery, Urology Division, Faculty of Medicine and Health Sciences, McGill University, Montreal, QC H3A 0G4, Canada; 3 The Research Institute, McGill University Health Centre, Montreal, QC H4A 3J1, Canada; 4 Department of Anatomy and Cell Biology, Faculty of Medicine and Health Sciences, McGill University, Montreal, QC H3A 0G4, Canada; 5 Department of Pharmacology and Therapeutics, Faculty of Medicine and Health Sciences, McGill University, Montreal, QC H3A 0G4, Canada

**Keywords:** lipid signalling, lysophosphatidic acid, LPA receptors, protein kinase C, PI3K, AKT substrates, spermatozoa

## Abstract

Lysophosphatidic acid (LPA) signalling is essential for maintaining germ cell viability during mouse spermatogenesis; however, its role in human spermatozoa is unknown. We previously demonstrated that peroxiredoxin 6 (PRDX6) calcium-independent phospholipase A_2_ (iPLA_2_) releases lysophospholipids such as LPA or arachidonic acid (AA) and that inhibiting PRDX6 iPLA_2_ activity impairs sperm cell viability. The exogenous addition of LPA bypassed the inhibition of PRDX6 iPLA_2_ activity and maintained the active phosphoinositide 3-kinase (PI3K)/AKT pathway. Here, we aimed to study PI3K/AKT pathway regulation via LPA signalling and protein kinases in maintaining sperm viability. The localization of LPARs in human spermatozoa was determined using immunocytochemistry, and P-PI3K and P-AKT substrate phosphorylations via immunoblotting. Sperm viability was determined using the hypo-osmotic swelling test. LPAR1, 3, 5 and 6 were located on the sperm plasma membrane. The inhibition of LPAR1-3 with Ki16425 promoted the impairment of sperm viability and decreased the phosphorylation of PI3K AKT substrates. Inhibitors of PKC, receptor-type PTK and PLC impaired sperm viability and the PI3K/AKT pathway. Adding 1-oleoyl-2-acetyl-snglycerol (OAG), a cell-permeable analog of diacylglycerol (DAG), prevented the loss of sperm viability and maintained the phosphorylation of PI3K. In conclusion, human sperm viability is supported by LPAR signalling and regulated by PLC, PKC and RT-PTK by maintaining phosphorylation levels of PI3K and AKT substrates.

## 1. Introduction

The mammalian spermatozoon faces numerous challenges in finding and fertilizing the oocyte in the oviduct. As spermatozoa traverse the female reproductive tract, they are exposed to different environments in the vagina, cervix, uterus and oviduct, and they must survive in order to reach the final destination and acquire fertilizing capacity through the process of sperm capacitation in the oviduct [1,2]. This could take up to 72 h, since the spermatozoon must wait for ovulation and complete the capacitation process. Thus, the spermatozoon must remain viable from ejaculation until it becomes capacitated, reaches the oocyte and fertilizes it.

Activating the phosphatidylinositol 3-kinase (PI3K)/protein kinase B (AKT) pathway is necessary to ensure human sperm viability [3]. The inhibition of the phosphorylation of PI3K or AKT is associated with the promotion of apoptotic-like changes, a decrease in sperm motility and increased lipid peroxidation and DNA oxidation, which ultimately leads to cell death [3,4]. The level of lipid oxidation, one of the apoptotic-like changes observed in spermatozoa, is regulated by peroxiredoxin 6 (PRDX6), an antioxidant enzyme with peroxidase activity that is highly expressed in human spermatozoa [5,6]. PRDX6 is unique to the family, demonstrating not only peroxidase but calcium-independent phospholipase A_2_ (iPLA_2_) and lysophosphatidylcholine acyl transferase (LPCAT) activities [7,8] as well. PRDX6 is vital in repairing oxidized cell membranes [9] in all three activities. We reported that knock-in male mice lacking only PRDX6 iPLA_2_ activity have high lipid peroxidation levels, thus demonstrating the importance of PRX6 in fighting against oxidative stress in spermatozoa [10,11].

Previously, we also found that PRDX6 iPLA_2_ activity maintains human sperm viability by activating the PI3K/AKT pathway through the release of fatty acids and lysophospholipids such as lysophosphatidic acid (LPA) and arachidonic acid (AA) [4]. The ablation of LPA receptors 1, 2 and 3 resulted in male mice with reduced sperm counts that became azoospermic as the males aged, thus suggesting that lysophosphatidic acid signalling is a pro-survival pathway during mouse spermatogenesis [12].

LPA is a bioactive lysophospholipid that plays an essential role in multiple cellular mechanisms such as cell proliferation and migration [13]; it exists in almost all mammalian cell types and is biosynthesized via two major metabolic pathways [14,15]. One of these pathways involves converting membrane phospholipids into phosphatidic acid with phospholipase D and then turning it into LPA species via phospholipase A1/A2(PLA_1/2_). The second way is by using PLA_1/2_, which cleaves membrane phospholipids into lysophospholipids, which metabolize into LPA species via phospholipase D [14]. LPA elicits a broad range of cellular mechanisms by acting on G protein-coupled receptors (GPCRs) called LPAR1-5 in the plasma membrane [13].

According to the Human Proteome Atlas (https://www.proteinatlas.org, accessed on 10 July 2023), LPAR1-4 is expressed in the human testes [16]. Mice testes highly express LPAR1-3 [12,17] and it has been demonstrated that these receptors are needed to support germ cell survival during spermatogenesis, as shown in the LPAR1-3 knock-out mouse model [12]. However, whether LPARs are present and the nature of the role of LPAR signalling in human spermatozoa are unknown. This study aimed to determine the presence of LPA receptors (LPAR) in human spermatozoa and whether LPAR signalling activates the PI3K/AKT pathway.

## 2. Materials and Methods

### 2.1. Materials

Rabbit polyclonal anti-phospho-PI3K (catalogue #4257S) and rabbit polyclonal anti-phospho-AKT substrate (catalogue #9611S) antibodies were bought from Cell Signaling (Beverly, MA, USA). Donkey polyclonal anti-rabbit (H+L) (catalogue #711-035-152) and goat polyclonal anti-mouse (H+L) (catalogue #115-035-062) antibodies, both conjugated with horseradish peroxidase, were purchased from Jackson Immuno Research Laboratories, Inc. (West Grove, PA, USA). The enhanced chemiluminescence (ECL) Kit (catalogue #32209), the goat polyclonal anti-rabbit (H+L) conjugated with AlexaFluor 555 (catalogue #A32732) and the Thermo Scientific protease inhibitor cocktail kit (catalogue #78410) were purchased from Thermo Fisher Scientific (Saint-Laurent, QC, Canada). Tyrphostin A47 was purchased from Calbiochem (San Diego, CA, USA). Percoll (catalogue #GE17-0891-01), rabbit polyclonal Anti-EDG2 (LPAR1, catalogue #SAB4500689), EDG7 (LPAR3, catalogue #SAB4501004), LPAR5 (catalogue #ABT114), LPAR6 (catalogue #HPA028934) and mouse monoclonal anti-α-tubulin (catalog #T5168) antibodies, Ki16425 (catalogue #SML0971), H-89 (catalogue #B1427), chelerythrine (catalogue #220285), PD98059 (catalogue #513000), PP2 (catalogue #P0042), U-73122 (catalogue #662035) and 1-Oleoyl-2-acetyl-sn-glycerol (OAG, catalogue #495414) were acquired from Sigma Aldrich (Oakville, ON, Canada). Other chemicals used were of at least reagent grade.

### 2.2. Sperm Preparation

Semen samples were collected from healthy volunteers (20–30 years old) by masturbation after three days of abstinence at the Royal Victoria Hospital (Montreal, QC, Canada) and incubated for 30 min at 37 °C to allow liquefaction. Liquefied semen was placed on a discontinuous Percoll gradient (20–40–65–95% Percoll layers) and centrifuged for 30 min at 2300× *g* and 20 °C. The highly motile spermatozoa recovered from the 95% layer and 65–95% interface were diluted to 50 × 10^6^ cell/mL in Biggers, Whitten and Whittingham (BWW) medium, pH 7.95 [18], in order to perform immunocytochemistry, or incubated with or without the following inhibitors for 3.5 h at 37 °C to evaluate the role of kinases in the regulation of sperm viability: 50 and 100 μM H89 (PKA inhibitor), 50 and 100 μM chelerythrine (PKC inhibitor), 150 and 300 μM PD98059 (MEK inhibitor), 0.5 and 1 μM PP2 (Src, Non-Receptor Tyrosine Kinase inhibitor), and 50 and 100 μM Tyrphostin A47 (EGFR, Receptor Tyrosine Kinase inhibitor), 2, 5, 10 and 20 μM Ki16425, an inhibitor of LPAR1-3, and 10 μM of U-73122, an inhibitor of PLC. The incubation time was selected as it is regularly used in our lab to capacitate spermatozoa under in vitro conditions.

### 2.3. Sperm Viability Determination

Sperm viability was determined using the hypo-osmotic swelling (HOS) test with modifications [19,20]. The treated sperm samples were gently mixed with 150 μL hypoosmotic (HOS) solution (1.5 mM fructose and 1.5 mM sodium citrate) after treatment and incubated at 37 °C for 30 min. The samples were then placed onto Superfrost plus slides. The sperm viability was evaluated with a Leica DFC 450C microscope at 200× magnification with Leica Application Suite X (LASX) software, Version1.1.0.12420 (Leica Microsystems, Wetzlar, Germany). Two hundred cells in duplicate were analyzed per sample and only viable spermatozoa showed different degrees of tail curling or the presence of a droplet [19].

### 2.4. Immunocytochemistry

The localization of LPA receptors in human spermatozoa was determined using immunocytochemistry. Washed spermatozoa were smeared onto Superfrost plus slides (Fisher Scientific, Montreal, QC, Canada) and permeabilized with 100% methanol for 10 min at −20 °C. One set of samples was not permeabilized in order to determine the labelling on the plasma membrane. Samples were rehydrated with PBS supplemented with Triton-X100 (PBS-T) and blocked with 5% goat serum in PBS-T/PBS for 30 min at 20 °C. Sperm smears were washed with PBS-T and incubated overnight at 4 °C with anti-LPAR1, LPAR3, LPAR5 or LPAR6 antibodies (1:50, 1:10, 1:10, and 1:10 dilution in 1% goat serum in PBS-T, respectively). The samples were then washed with PBS-T and incubated with goat anti-rabbit (H+L) antibody conjugated with AlexaFluor 555 (1:2000 dilution in PBS-T + 1%BSA) for 1 h at 20 °C. A negative control was prepared by omitting the primary antibody to confirm the absence of non-specific binding. Pictures showing LPARs labelling were taken using a Carl Zeiss Axiophot microscope (Oberkochen, Germany) at 100× magnification.

### 2.5. SDS-PAGE and Immunoblotting

After treatments, sperm samples were supplemented with an electrophoresis buffer containing 100 μM vanadate, 20 mM β-glycerolphosphate, 5 mM sodium fluoride with 100 mM dithiothreitol and a proteinases inhibitor cocktail in order to evaluate the phosphorylation of PI3K (P-PI3K) and AKT substrates (P-ATK-S) using SDA-PAGE and immunoblotting. The sperm proteins in the electrophoresis buffer were boiled at 100 °C for 5 min and centrifuged at 21,000× *g* for 5 min at 20 °C. The supernatant containing the sperm proteins was then loaded onto 10% polyacrylamide gels, electrophoresed and electrotransferred onto nitrocellulose for 45 min. The membranes were blocked for 1 h in 5% skimmed milk in 0.1% Tween 20 in 2 mM Tris buffer saline (TTBS) before incubation with anti-LPAR1, anti-LPAR6, anti-p-PI3K and anti-p-AKT substrate antibodies (1:1000, 1:1000, 1:2000 and 1:1000 in antibody buffer (TBS 1×, 0.1% Tween 20, 25 mg/mL BSA and ddH_2_O), respectively) overnight at 4 °C. The following day, the membranes were washed with TTBS 1×. They were then incubated with the donkey anti-rabbit secondary antibody (1:2500 diluted in TTBS) for 45 min at room temperature and washed with TTBS 1×. Finally, the membranes were soaked in ECL solution for 5 min before detecting positive immunoreactive bands using Amersham Imager 600 (GE Healthcare, Chicago, IL, USA). The relative intensities of protein bands per sample were assessed using Fiji Image J (National Institutes of Health, Stapleton, NY, USA) and standardized to that of α-tubulin or silver stain (the loading control) by dividing the relative intensity of the protein band of interest. The relative intensities were then normalized to the respective control and the average relative intensity and standard error were subsequently determined for each experiment.

### 2.6. Statistical Analysis

All graphical results were presented as mean ± SEM. The Shapiro–Wilk and Levene’s tests were used to assess normal data distribution and variance homogeneity, respectively. We used the one-way ANOVA and the Dunnett’s test to determine the statistical differences among the groups (*p* ≤ 0.05).

## 3. Results

### 3.1. LPAR Are Differentially Located in Human Spermatozoa and Are Active in Supporting Sperm Viability

**Figure 1:**
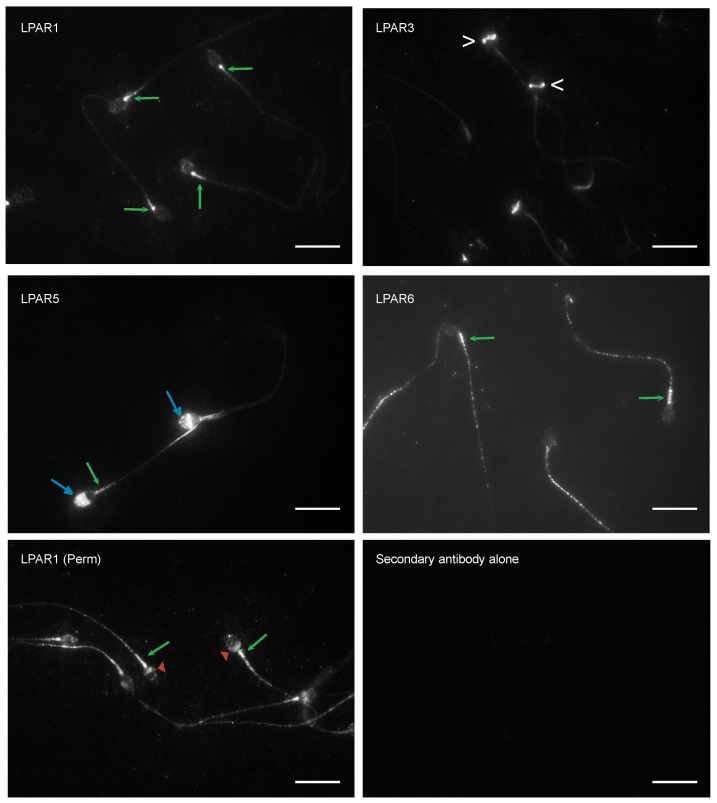
Localization of LPARs in human spermatozoa. Human spermatozoa, non-permeabilized or permeabilized with 100% methanol (Perm), were incubated with anti-LPAR antibodies. Spermatozoa incubated with the second one alone did not show any signals. The green arrow indicates the sperm midpiece; the white arrowhead indicates the sperm equatorial segment; the light blue arrow indicates the sperm acrosome; and the red head arrow indicates the post-acrosomal region. Pictures were taken at 100× using a Carl Zeiss Axiophot microscope (Oberkochen, Germany), scale bar = 15 μm.

The addition of LPA prevented the decrease in sperm viability when PRDX6 iPLA_2_ activity was inhibited, suggesting the participation of LPA signalling as a pro-survival pathway in human spermatozoa [4]. First, we wanted to know whether human spermatozoa have LPA receptors. Therefore, we determined the location of LPARs on human ejaculated spermatozoa using immunocytochemistry. We found that the labelling of the four LPARs investigated was detected in the midpiece and flagellum of human spermatozoa (Figure 1). LPAR1 was present in permeabilized and non-permeabilized (Perm) human sperm, but LPAR3, LAPR5 and LPAR6 were only observed in non-permeabilized spermatozoa. Interestingly, LPAR1 was also observed in the post-acrosome and midpiece regions in permeabilized human spermatozoa, and LPAR3 and LPAR5 were found in the equatorial segment and acrosome region, respectively, in non-permeabilized human spermatozoa.

**Figure 2:**
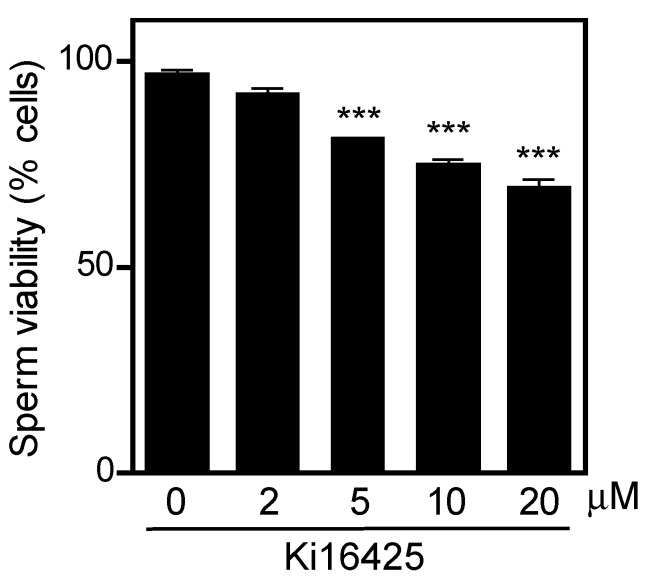
The effect of Ki16425 causes the cell death of human spermatozoa. Spermatozoa were treated with Ki16425 for 3.5 h at 37 °C and a hypo-osmotic swelling buffer was added to distinguish whether the spermatozoa were non-viable or viable (Section 2). The results are representative of sperm samples from different healthy donors (n = 4; *** *p* ≤ 0.001, ANOVA and Dunnett’s test).

**Figure 3:**
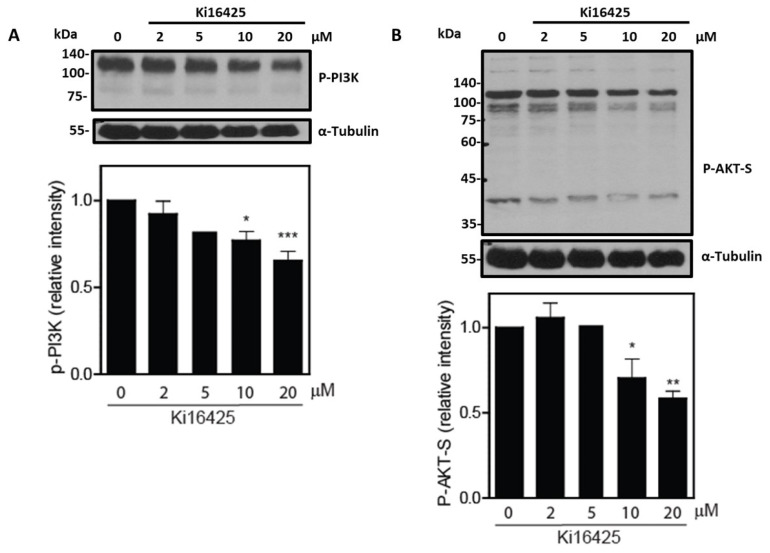
The effect of Ki16425 on PI3K and AKT substrate phosphorylation of human spermatozoa. Spermatozoa were treated with increasing concentrations of Ki16425 for 3.5 h at 37 °C. Sperm proteins were electrophoresed, electrotransferred and immunoblotted with (**A**) anti-P-PI3K or (**B**) anti-P-AKT-S antibodies. The membrane was stripped and reblotted with the anti-Tubulin antibody to confirm equal loading for each lane. The relative intensity of P-PI3K/P-AKT-S was obtained by normalizing each band’s intensity to the respective intensity of tubulin. The results are representative of sperm samples from different healthy donors (n = 4; * *p* ≤ 0.05, ** *p* ≤ 0.01, *** *p* ≤ 0.001, ANOVA and Dunnett’s test).

Sperm survival is associated with the activation of the PI3K/AKT pathway [3]. Thus, we wanted to know whether LPAR action is necessary to activate the PI3K/AKT pathway in order to sustain viability in human spermatozoa. When spermatozoa were treated with Ki16425, an inhibitor of LPAR1-3, we found a dose-dependent decrease in sperm viability and PI3K and AKT substrates’ phosphorylations compared to non-treated cells (Figure 2 and Figure 3). These data suggest the presence of active LPAR signalling to activate the PI3K/AKT pathway in spermatozoa.

### 3.2. The Inhibition of Different Kinases Promotes the Loss of Phosphorylated PI3K and AKT Substrates and Leads to a Decrease in Sperm Viability

To decipher the regulation of the survival pathway in spermatozoa, we explored whether different kinases (protein kinase A (PKA) and protein kinase C (PKC), extracellular signal-regulated kinase (ERK) and non-receptor type protein tyrosine kinase (NR-PTK) and receptor-type protein tyrosine kinase (R-PTK)), known to be active in human spermatozoa to sustain motility and capacitation [21,22,23], are involved in this regulation. We incubated spermatozoa with H89, chelerythrine, PD98059, PP2 and Tyrphostin A47, inhibitors of PKA, PKC, MEK, NR-PTK and R-PTK, respectively, for 3.5 h at 37 °C. This time was chosen as it is the established period to promote capacitation in human spermatozoa under in vitro conditions [24]. This strategy was appropriate for studying the viability regulation in spermatozoa incubated during a time similar to the one needed for capacitation.

**Figure 4:**
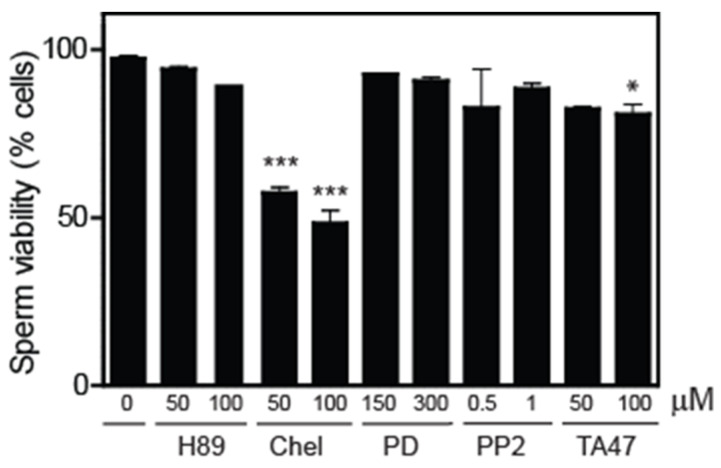
The effect of kinase inhibitors caused the cell death of human spermatozoa. Spermatozoa were treated with kinase inhibitors for 3.5 h at 37 °C. Then, the hypo-osmotic swelling buffer was added to distinguish whether the sperm was non-viable or viable (Section 2) (n = 3, which means a significant difference within the same strain). The results are representative of sperm samples from different healthy donors (ANOVA and Dunnett’s test; * *p* ≤ 0.05; *** *p* ≤ 0.001).

**Figure 5:**
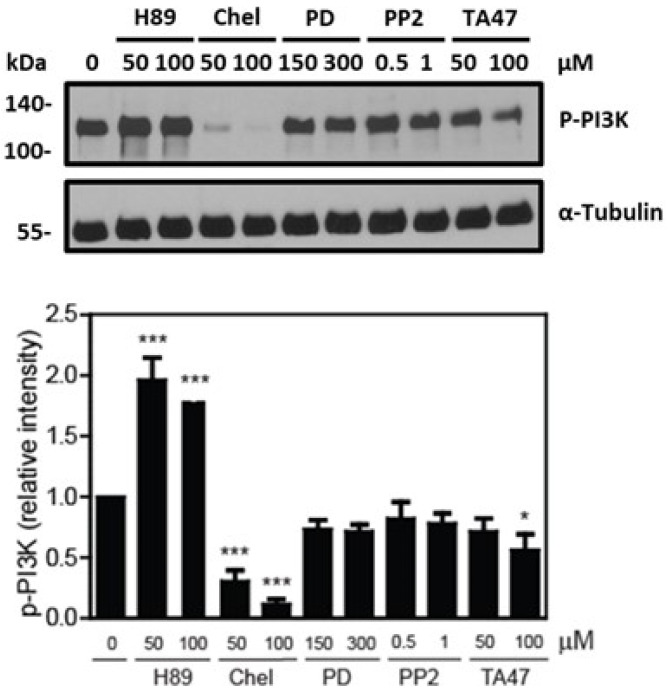
The different kinase inhibitors affected the PI3K phosphorylation of human spermatozoa. Levels of P-PI3K were determined in spermatozoa incubated with H89, chelerythrine, PD98059, PP2 and Tyrphostin A47 for 3.5 h at 37 °C. Sperm proteins were electrophoresed, electrotransferred and immunoblotted with the anti-P-PI3K antibody. The membrane was stripped and reblotted with the anti-Tubulin antibody to confirm equal loading for each lane. The relative intensity of P-PI3K was obtained by normalizing each band’s intensity to the respective intensity of tubulin. The results are representative of sperm samples from different healthy donors (n = 4; * *p* ≤ 0.05, *** *p* ≤ 0.001, ANOVA and Dunnett’s test).

**Figure 6:**
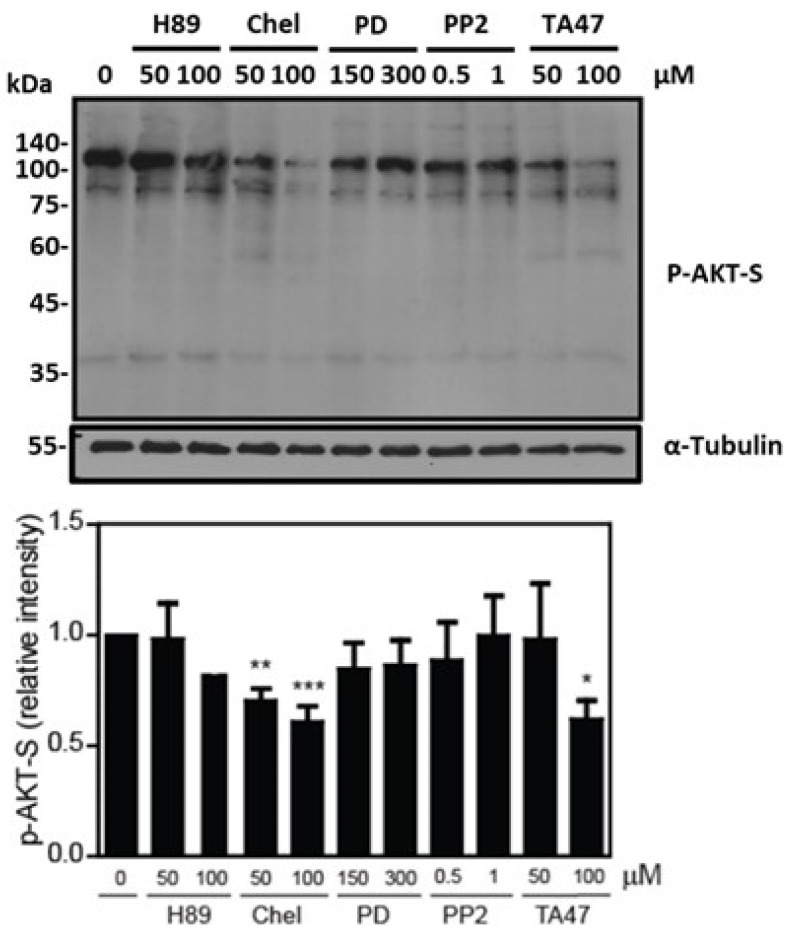
The different kinase inhibitors led to the effect on AKT substrates’ phosphorylation of human spermatozoa. Levels of P-AKT substrates were determined in spermatozoa incubated with H89, chelerythrine, PD98059, PP2 and Tyrphostin A47 for 3.5 h at 37 °C. Sperm proteins were electrophoresed, electrotransferred and immunoblotted with the anti-P-AKT-S antibody. The membrane was stripped and reblotted with the anti-Tubulin antibody to confirm equal loading for each lane. The relative intensity of P-AKT-S was obtained by normalizing each band’s intensity to the respective intensity of tubulin (n = 4; * *p* ≤ 0.05 ** *p* ≤ 0.01 *** *p* ≤ 0.001, ANOVA and Dunnett’s test).

We observed that chelerythrine promoted the highest impairment of sperm viability and a lesser but significant reduction in Tyrphostin A47-treated spermatozoa compared to non-treated controls (Figure 4). The phosphorylation of PI3K and AKT substrates decreased in spermatozoa treated with Chelerythrine or tyrphostin A47 compared to untreated controls (Figure 5 and Figure 6). Sperm viability was not impaired by the other kinase inhibitors, suggesting that PKC and RT-PTK are sufficient to maintain viability in human spermatozoa. Based on these findings, we further explored the regulatory mechanisms necessary to support sperm viability.

**Figure 7:**
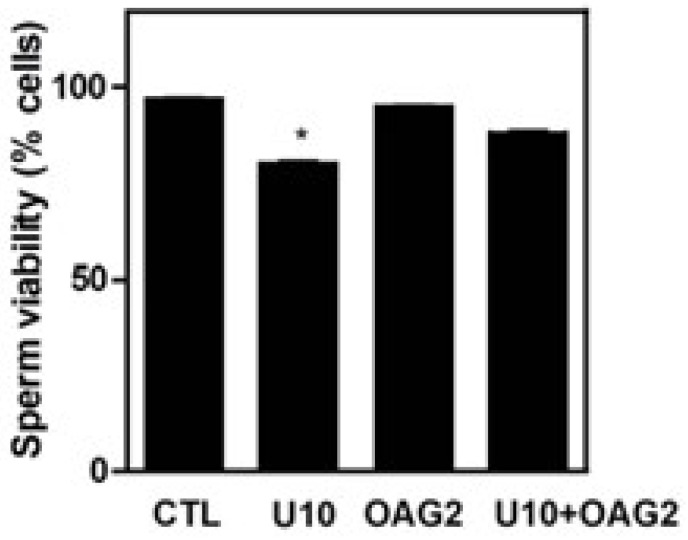
OAG prevents the loss of human sperm viability when U-73122 is present. Spermatozoa were incubated with U-73122 and OAG for 3.5 h at 37 °C and a hypo-osmotic swelling buffer was added to distinguish whether the spermatozoa were non-viable or viable (Section 2). The results are representative of sperm samples from different healthy donors (n = 3 * means a significant difference within the same strain. ANOVA and Dunnett’s test; * *p* ≤ 0.05).

**Figure 8:**
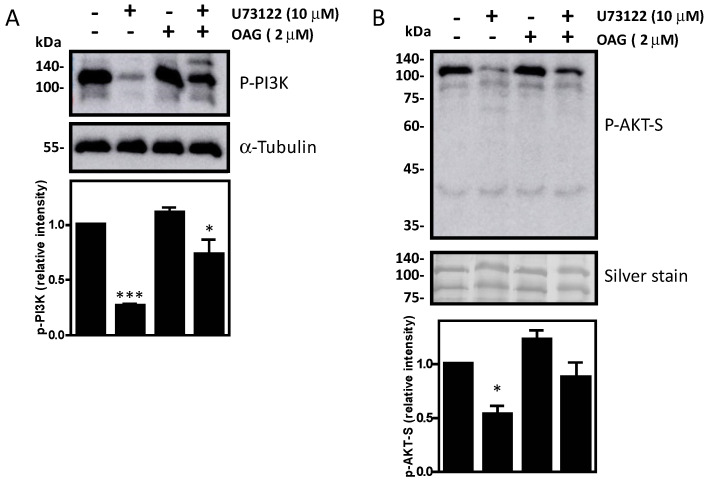
OAG prevents the loss of PI3K and AKT substrates’ phosphorylation when U−73122 is present. Levels of P-PI3K and P-AKT-S in spermatozoa incubated with U-73122 and OAG for 3.5 h at 37 °C. Sperm proteins were electrophoresed, electrotransferred and immunoblotted with the anti-PI3K/P-AKT-S antibody. The membrane was stripped and reblotted with the anti-Tubulin antibody to confirm equal loading for each lane. The relative intensity of (**A**) P-PI3K and (**B**) P-AKT-S was obtained by normalizing each band’s intensity to the respective intensity of tubulin. The results are representative of sperm samples from different healthy donors (n = 4, * means significant difference within the same strain. ANOVA and Dunnett’s test; * *p* ≤ 0.05; *** *p* ≤ 0.001).

The phospholipase C/PKC pathway is active during sperm capacitation and acrosome reaction in human spermatozoa [25]. To further characterize the PKC pathway concerning the maintenance of sperm viability, we assessed whether 1-Oleoyl-Oleoyl-2-acetylacetylacetylacetyl-sn-glycerol (OAG), a permeable analog of diacylglycerol known to activate PKC, prevented the impairment of sperm viability caused by the inhibition of PLC activity with U-73122 (Figure 7). Adding OAG to the incubation medium prevented the inhibition of PLC by U-73122 in spermatozoa. Moreover, OAG prevented the decrease in PI3K and AKT substrates’ phosphorylation due to PLC inhibition (Figure 8).

## 4. Discussion

**Figure 9:**
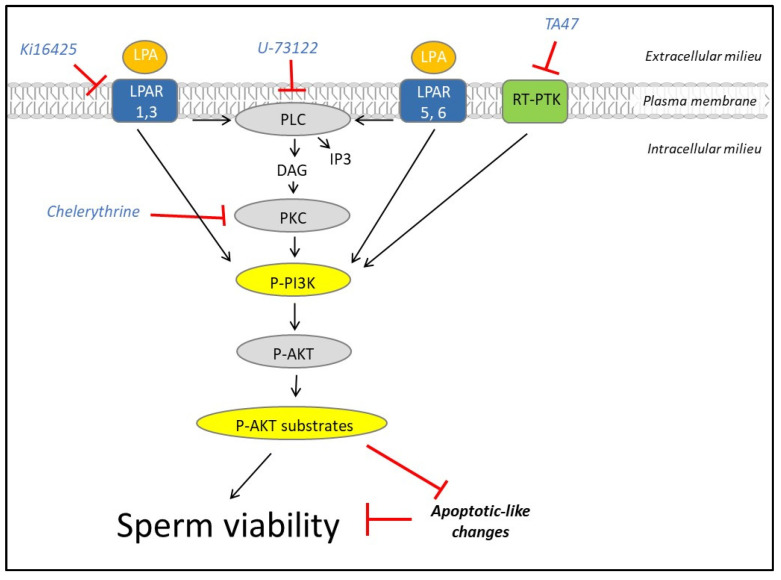
Lysophosphatidic signalling is essential to support viability in human spermatozoa. Lysophosphatidic acid (LPA) binds to LPAR (1, 3, 5 or 6) to activate the PI3K/AKT pathway in human spermatozoa to prevent apoptotic-like changes that could impair sperm viability. Ki6425 inhibits LPAR1 and LPAR3, thus preventing phosphorylations of PI3K and AKT substrates and leading to impairment of sperm viability. A similar fate occurs in spermatozoa treated with U−73122, TA47 or chelerythrine, inhibitors of PLC, receptor-type tyrosine-protein kinase (RT-PTK) and PKC, respectively.

The results presented in this report demonstrate, for the first time, that LPAR1, LPAR3, LPAR5 and LPAR6 are present in ejaculated human spermatozoa and that active LPA signalling is involved in the maintenance of sperm viability and is regulated mainly by PKC and, to a lesser extent, by RT-PTK (Figure 9).

LPARs are mainly located in the plasma membrane. Still, their differential expression in sperm compartments supports their important role in maintaining cell viability and possible participation in other essential functions of human spermatozoa. LPAR1, 3, 5 and 6 are expressed in the plasma membrane of the flagellum and midpiece regions of the human spermatozoon (Figure 1). These findings indicate a wide distribution of LPARs needed to sustain sperm viability.

LPAR3 is located in the equatorial region (Figure 1); thus, we can hypothesize its involvement in the sperm–egg fusion process, since the plasma membrane of the equatorial segment remains in the spermatozoon after the acrosome reaction and it is the place where the fusion with the oolemma occurs. The finding of LPAR5 in the acrosomal region coincides with the location of SLC9A3 (also known as NHE3), a Na^+^/H^+^ exchanger found in elongating spermatids and critical for acrosomal formation during spermiogenesis [26]. SLC9A3 is involved in the acidification of the epididymal fluid [27]. During human sperm capacitation, the acrosome is alkalinized [28]. Therefore, it is possible that LPAR5 not only participates in the formation of the acrosome during spermiogenesis, but has a role in ejaculated spermatozoa by participating in the alkalinization of the acrosome during capacitation in preparation for the acrosome reaction. Further studies are needed to confirm this possibility.

LPAR1 is also located in the acrosomal region and midpiece of permeabilized spermatozoa (Figure 1). It was previously demonstrated that PI3K signalling is involved in the human sperm acrosome reaction [29]. Knowing from the current study that LPAR1 activates PI3K to support viability, it is also possible that LPRA1 activates PI3K during the acrosome reaction.

The strong signal of LPAR1 in the post-acrosomal region of permeabilized spermatozoa indicates a possible association with the perinuclear theca [30]. This structure plays a vital role during fertilization [30,31]. There is strong evidence that LPAR1 is expressed in the nucleus of different cell types, such as the pheochromocytoma of the rat adrenal medulla (PC12), human bronchial epithelial cells (HBEC), rat liver cells and rat hepatoma cells (HTC4) [32,33,34]. However, the role of LPAR1 associated with the sperm perinuclear theca or nucleus is currently unknown.

Recently, we reported that PRDX6 calcium-independent phospholipase A_2_ (iPLA_2_) regulated the PI3K/AKT pathway and that the addition of LPA or AA prevented the impairment of sperm viability due to the inhibition of PRD6 iPLA_2_ activity [4]. Here, we provided evidence for active LPA signalling to regulate the PI3K/AKT pathway to maintain sperm viability. Our results established the importance of LPARs in maintaining human sperm survival, since the presence of Ki16425 in the incubation medium significantly reduces sperm viability compared to untreated controls (Figure 3). Although Ki16425 is a competitive antagonist of LPAR1, 2 and 3, it significantly affects LPAR1 and LPAR3 but not LPAR2 [35]. We can conclude that LPAR1 and LPAR3 are associated with maintaining sperm viability, as Ki6425 impaired the phosphorylation of PI3K and AKT substrates. Mice lacking LPAR1, LPAR2 and LPAR3 have reduced sperm production due to the increased death of germ cells in the testes [12]. Thus, LPAR1 and LPAR3 are essential to maintain sperm viability and ensure male fertility. These findings in mice testes [12] and human spermatozoa demonstrate the need for LPA signalling to support the survival of germ cells during spermatogenesis and to ensure the viability of the ejaculated spermatozoa in the female genital tract.

LPAR signalling participates in a broad range of cellular mechanisms, depending on the activation of the downstream pathways via the activation of various G proteins, such as Rho, MAPK, PKC and PI3K [13]. The impairment of viability and the inhibition of the phosphorylation of PI3K and P-AKT substrates observed in chelerythrine-treated spermatozoa compared to untreated controls support the essential role of PKC in maintaining human spermatozoa viability (Figure 4, Figure 5 and Figure 6). PKC is activated by diacylglycerol (DAG), a second messenger produced by phospholipase C (PLC) that has been shown to initiate further signal transduction pathways by PKC [36]. PLC signalling participates in spermatozoa in various processes such as thermotaxis, sperm capacitation and acrosome reaction [25,37,38,39]. We found that the inhibition of PLC with U-73122 leads to decreased sperm viability (Figure 7). The exogenous addition of OAG, a permeable DAG analog, bypassed the inhibition and maintained high phosphorylation levels of PI3K and AKT substrates similar to those observed in untreated controls (Figure 8). These findings indicate that PLC signalling activates PKC to maintain high phosphorylation levels of PI3K and AKT substrates to sustain viability in human spermatozoa.

The less decreased and yet statistically significant levels of sperm viability observed in Tyrphostin A47-treated spermatozoa suggest a role of RT-PTK. EGFR is an RT-PTK found in human spermatozoa and is involved in regulating sperm motility [40]. The reduction in sperm viability and phosphorylation levels of PI3K and AKT substrates by Thyrsphostin A47 indicates the participation of EFG in the maintenance of sperm survival. Based on these results, we can suggest the existence of crosstalk between the PLC and EGF pathways to ensure high phosphorylation levels of PI3K and AKT substrates to maintain viability in human spermatozoa.

## 5. Conclusions

For the first time, we demonstrated the presence of LPAR1, LPAR3, LPAR5 and LPAR6 in human spermatozoa and the need for active LPA signalling to ensure sperm viability. This study strongly supports the role of PKC as an essential regulator of the LPAR-PI3K-AKT pathway to maintain sperm viability in humans. There is crosstalk between PLC and EFG signalling to ensure the survival of human spermatozoa. Further research is needed to elucidate molecular mechanisms associated with the interaction between EGFR and LPAR and to establish other functions of LPARs in human spermatozoa.

## Data Availability

The data that support the findings of this study are available from the corresponding author upon reasonable request.
